# Supplementary material for: Seed Priming with MeJa Prevents Salt-Induced Growth Inhibition and Oxidative Damage in Sorghum bicolor by Inducing the Expression of Jasmonic Acid Biosynthesis Genes
Source: Int J Mol Sci. 2023 Jun 20;24(12):10368. doi: 10.3390/ijms241210368 (PMC10299227; doi:10.3390/ijms241210368)
Supplement: Supplementary file 1 [file ijms-24-10368-s001.zip › ijms-2378855-supplementary.pdf]

International Journal of
Molecular Sciences

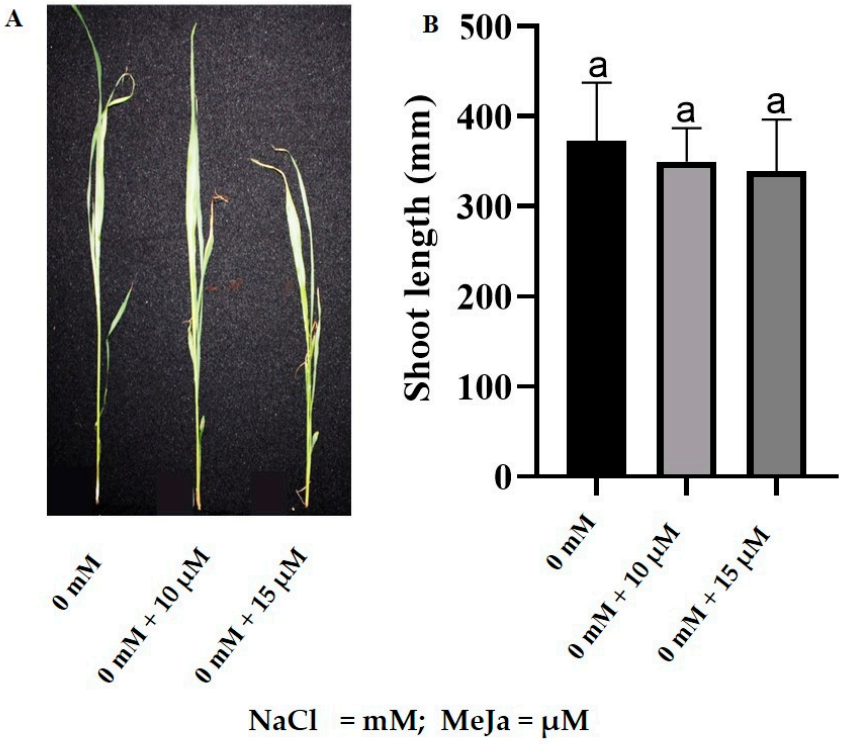

Figure S1. Effect of Methyl Jasmonate (MeJa) on the growth of *S. bicolor* under normal condition. Phenotype (**A**) and shoot length (**B**) of *S. bicolor* plants grown from seeds primed with 10 and 15 µM MeJa in the absence of salt stress. Data in the figure represent the mean ± standard deviation from three biological replicates. Different letters indicate significant differences ($p < 0.05$) based on ANOVA one-way variance analysis following Tukey's comparison test.

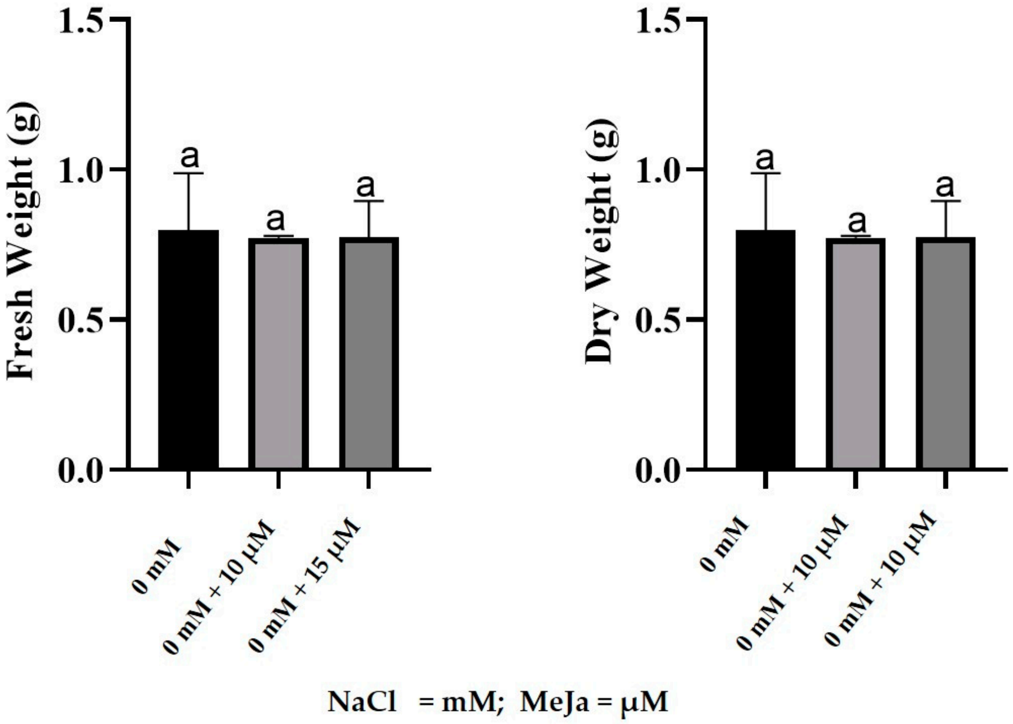

Figure S2. Effect of Methyl Jasmonate (MeJa) on biomass of *S. bicolor* under normal conditions. Fresh weight (**A**) and dry weight (**B**) of *S. bicolor* plants grown from seeds primed with 10 and 15 µM MeJa in the absence of salt stress. Data in the figure represent the mean ± standard deviation from three biological replicates. Different letters indicate significant differences ($p < 0.05$) based on ANOVA one-way variance analysis following Tukey's comparison test.

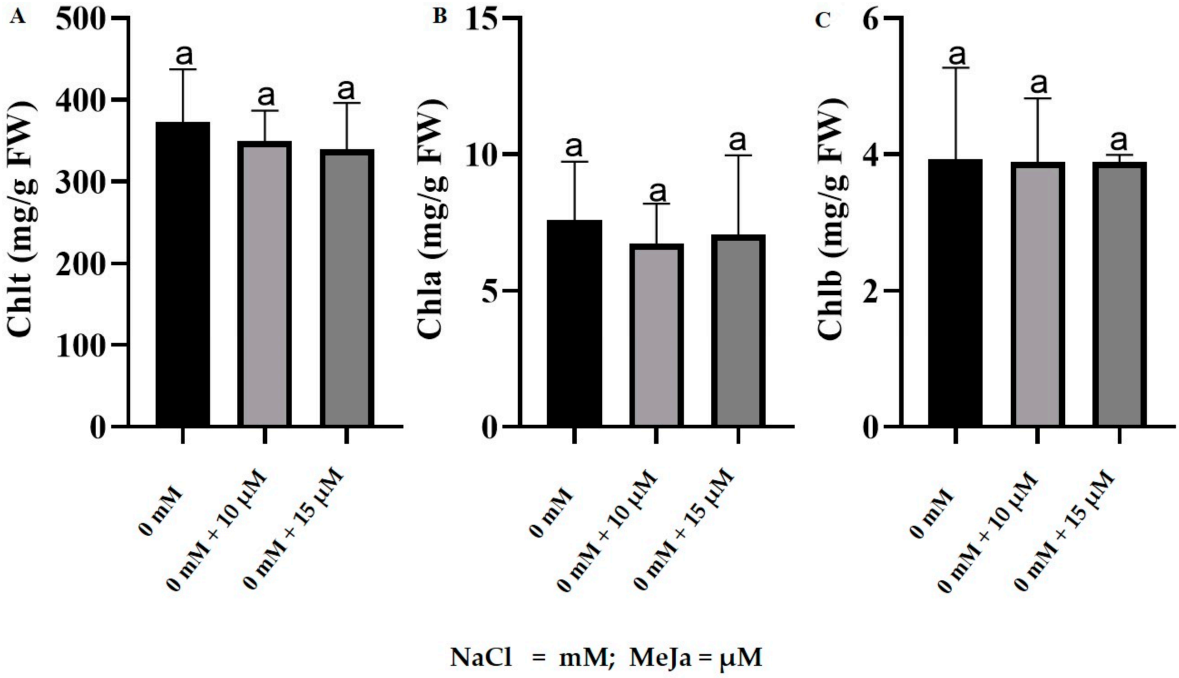

Figure S3. Effect of Methyl Jasmonate (MeJa) on the chlorophyll content in *S. bicolor* under normal (non-stressed) conditions. Photosynthetic pigments assayed include Chlorophyll *a* (**A**), Chlorophyll *b* (**B**) and total Chlorophyll (**C**) were determined from *S. bicolor* plants grown from seeds primed with 10 and 15 µM MeJa and stressed with 100 and 200 mM NaCl. Data in the table represent the mean ± standard deviation from three biological replicates ($n = 3$). Different letters in the column indicate significant differences ($p < 0.05$) based on ANOVA one-way variance analysis using Tukey's comparison test.

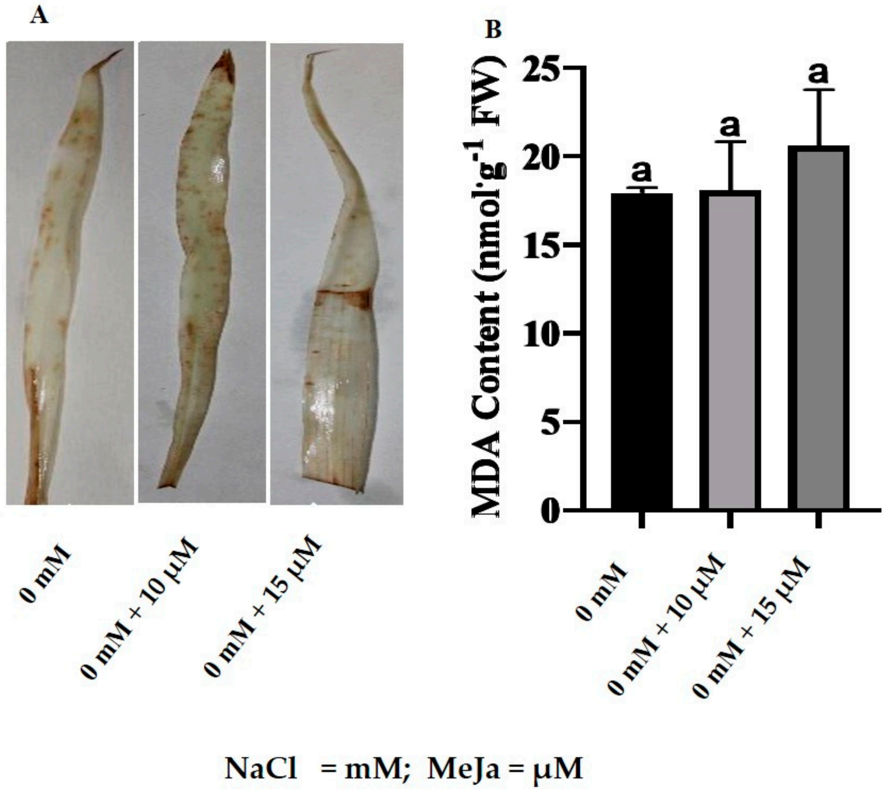

Figure S4. Assessment of oxidative damage in *S. bicolor* in response to MeJa under normal (non-stressed) conditions. Accumulation of H_2O_2 on the leaves was determined by histochemical staining (**A**), and lipid peroxidation was determined by measuring MDA content (**B**) for *S. bicolor* plants grown from seeds primed with 10 and 15 μM MeJa. Data in the figure represent the mean ± standard deviation from three biological replicates. Different letters indicate significant differences ($p < 0.05$) based on ANOVA one-way variance analysis using Tukey's comparison test.

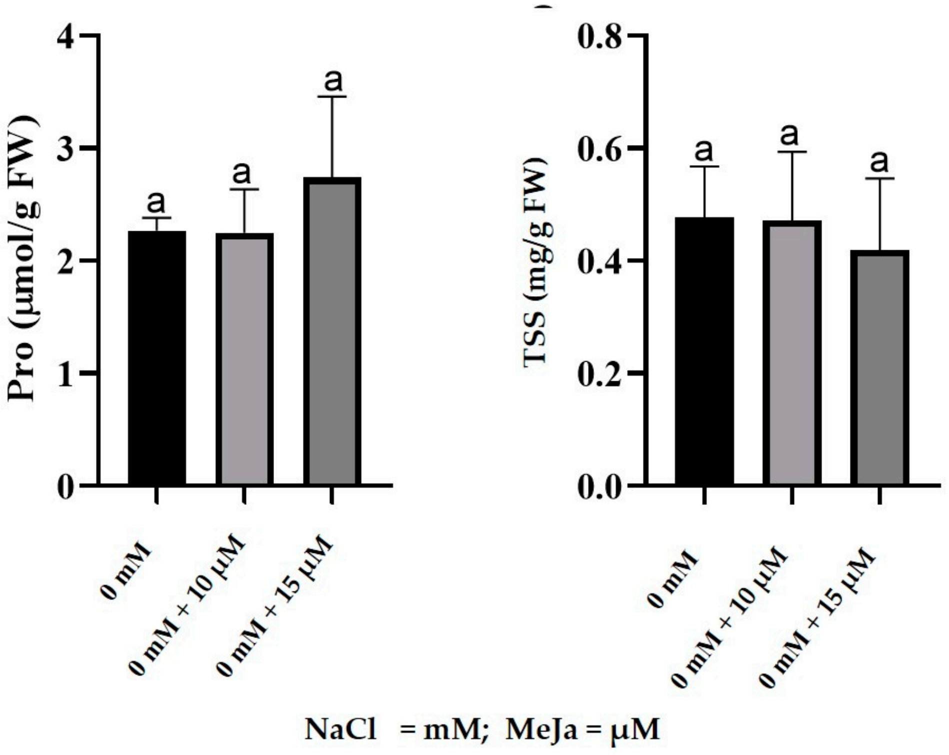

Figure S5. Proline (Pro) and total soluble sugar (TSS) content in response to MeJa pre-treatment in *S. bicolor* under normal (non-stressed) conditions. Proline content in *S. bicolor* plants grown from seeds primed with 10 and 15 µM MeJa under 100 mM NaCl (**A**) or 200 mM NaCl (**B**). Total soluble sugars content in *S. bicolor* plants pre-treated with 10 µM or 15 µM MeJa under 100 mM NaCl (C) or 200 mM NaCl (D). Data in the figure represent the mean ± standard deviation from three biological replicates. Different letters indicate significant differences ($p < 0.05$) based on ANOVA one-way variance analysis using Tukey's comparison test
